# Can Alkyl Quaternary Ammonium Cations Substitute H_2_O_2_ in Controlling Cyanobacterial Blooms—Laboratory and Mesocosm Studies

**DOI:** 10.3390/microorganisms9112258

**Published:** 2021-10-29

**Authors:** Xinya Zhang, Yiruo Xia, Yunlu Jia, Assaf Sukenik, Aaron Kaplan, Chanyuan Song, Guofei Dai, Fang Bai, Lin Li, Lirong Song

**Affiliations:** 1 State Key Laboratory of Freshwater Ecology and Biotechnology, Institute of Hydrobiology, Chinese Academy of Sciences, Wuhan 430072, China; xinyazhang0423@hotmail.com (X.Z.); xiayiruo19@mails.ucas.ac.cn (Y.X.); songcy7722@163.com (C.S.); fangbai@ihb.ac.cn (F.B.); lilin@ihb.ac.cn (L.L.); 2 College of Advanced Agricultural Sciences, University of Chinese Academy of Sciences, Beijing 100049, China; 3 The Yigal Allon Kinneret Limnological Laboratory, Israel Oceanographic and Limnological Research, P.O. Box 447, Migdal 14950, Israel; assaf@ocean.org.il; 4 Department of Plant and Environmental Sciences, Edmond J. Safra Campus, The Hebrew University of Jerusalem, Givat Ram, Jerusalem 9190401, Israel; aaron.kaplan@mail.huji.ac.il; 5 Jiangxi Provincial Key Laboratory of Water Resources and Environment of Poyang Lake, Jiangxi Institute of Water Sciences, Nanchang 330029, China; daiguofei1985@126.com

**Keywords:** cyanocide, *Microcystis*, cationic surfactant, inactivation, aquaculture

## Abstract

Mitigation of harmful cyanobacterial blooms that constitute a serious threat to water quality, particularly in eutrophic water, such as in aquaculture, is essential. Thus, in this study, we tested the efficacy of selected cyanocides towards bloom control in laboratory and outdoor mesocosm experiments. Specifically, we focused on the applicability of a group of cationic disinfectants, alkyltrimethyl ammonium (ATMA) compounds and H_2_O_2_. The biocidal effect of four ATMA cations with different alkyl chain lengths was evaluated ex situ using *Microcystis* colonies collected from a fish pond. The most effective compound, octadecyl trimethyl ammonium (ODTMA), was further evaluated for its selectivity towards 24 cyanobacteria and eukaryotic algae species, including Cyanobacteria, Chlorophyta, Bacillariophyta, Euglenozoa and Cryptophyta. The results indicated selective inhibition of cyanobacteria by ODTMA-Br (C18) on both Chroccocales and Nostocales, but a minor effect on Chlorophytes and Bacillariophytes. The efficacy of ODTMA-Br (C18) (6.4 μM) in mitigating the *Microcystis* population was compared with that of a single low dose of H_2_O_2_ treatments (117.6 μM). ODTMA-Br (C18) suppressed the regrowth of *Microcystis* for a longer duration than did H_2_O_2_. The results suggested that ODTMA-Br (C18) may be used as an effective cyanocide and that it is worth further evaluating this group of cationic compounds as a treatment to mitigate cyanobacterial blooms in aquaculture.

## 1. Introduction

Excess input of nitrogen, phosphorus and other nutrients make cyanobacteria proliferate exceptionally. The massive accumulation of cyanobacterial biomass can damage water quality [1]. Some cyanobacteria are known to produce toxic secondary metabolites, such as microcystins, which can be harmful to zooplankton, fish and even humans [2,3]. In fish aquaculture systems, extensive feeding protocols lead to water hyper-eutrophication that facilitates frequent and persistent occurrence of cyanobacterial harmful algal blooms (cyanoHABs). Continuous pond aeration is usually required to eliminate hypoxia or anoxic conditions resulting from large amounts of photosynthetic and respiratory activities (respectively) caused by large water blooms, as well as the associated additional costs in aquaculture production [4]. Therefore, effective prevention of cyanobacterial blooms and domination of eukaryotic algae is an important and challenging task in aquaculture.

Several strategies have been developed to mitigate cyanobacterial blooms, including physical, chemical and biological methods [5]. Each strategy has its own advantages and disadvantages. Physical methods, e.g., mechanical harvest [6], flocculation and sedimentation [7], light shading [8] and ultrasonic technology [9], are often costly and show limited efficacy. Chemical methods, e.g., copper-based compounds [10], herbicidal agents [10] and peroxides, such as H_2_O_2_ [11], are commonly used. H_2_O_2_ has been used as a benign chemical to decrease cyanobacteria as it decomposes into water and oxygen without chemical residues [12]. Moreover, H_2_O_2_ may have a more pronounced effect on cyanobacteria, which are prokaryotic, than on the eukaryotic phytoplankton [11,12,13]. Matthijs et al. [11] studied the influence of H_2_O_2_ on the toxic cyanobacterium *Planktothrix agardhii* in Lake Koetshuis, the Netherlands, and found that 2.0 mg/L H_2_O_2_ could prevent the excessive multiplication of *Planktothrix agardhii* in the surface water; meanwhile, there was little effect on the eukaryotic phytoplankton (including green algae, cryptophytes, chrysophytes and diatoms), zooplankton and macrofauna. However, H_2_O_2_ can be only used in short-term applications, as H_2_O_2_ would be rapidly consumed and degraded in the water column [14]. Biological approaches are intended to alter the ecosystem towards less favorable conditions for cyanobacteria. However, many of these methods have limited effects on algae control, such as the use of competing microorganisms and fish introduction [15,16]. Among these strategies, the application of cyanocides is considered a rapid and affordable approach to control the growth of cyanobacteria and their bloom [17]. Particularly, the application of chemical methods is routinely used to control toxic cyanoHABs [5].

Quaternary ammonium compounds (QACs) possess strong algaecide properties and are widely used to maintain pool water quality [18]. Due to their amphiphilic nature [19,20], QACs bind to and denature membrane proteins and thereby affect the integrity of cell membranes. Electrostatic interactions between the positively charged QAC head and the negatively charged bacterial cell membrane, with penetration of the QAC side chain into the intra-membrane region, eventually leads to leakage of cytoplasmic material and cell lysis [20,21]. Recently, two studies [21,22] showed that low concentrations of the octadecyltrimethylammonium (ODTMA) cation inhibited photosynthesis and destroyed cells of two different cyanobacteria *Microcystis* and *Aphanizomenon*. In this case, Sukenik’s team used two columns in series, each consisting of 10 g of granular ODTMA-clay complex mixed with 650 g of sand [22]. In addition, Wu’s team noted that cyanobacterial cells (*Aphanizomenon* or *Microcystis*) disintegrate and lose their metabolic activity (photosynthesis) when exposed to ATMA bromide, with ED50 (1 h) of ODTMA-Br (C18) ranging between 1.5 and 7 μM [21]. The current study further assessed the selectivity of the ODTMA cation toward different phytoplankton taxa and the feasibility of using ODTMA as a cyanocide in aquaculture. We confirm the cyanocidal effect of alkyl trimethyl ammonium (ATMA) compounds and the importance of the length of the alkyl chain towards photosynthesis inhibition of *Microcystis* colonies collected from aquaculture ponds. Furthermore, the algaecidal spectrum of ATMA compounds was evaluated using several cyanobacteria and eukaryotic algae. An effective dose of ODTMA-Br (C18) (6.4 μM), determined in laboratory studies, was used in mesocosm experiments to examine its selectivity towards cyanobacteria but not eukaryotic algae and extended effect on cyanobacterial growth. The efficiency and selectivity of ODTMA-Br (C18) toward cyanobacteria growth were compared with that of H_2_O_2_ (117.6 μM) application.

## 2. Materials and Methods

### 2.1. Organisms and Materials

All cyanobacteria and eukaryotic algae used in the laboratory experiments were obtained from Culture Collection of the Freshwater Algae of the Institute Hydrobiology (FACHB-Collections, Wuhan, China). The cells were cultivated in a BG-11 medium [23] at 25 °C and a light/dark cycle of 12/12 h. The light intensity was 30 μmol m^−2^ s^−1^ provided by fluorescent lamps (Philips, The Netherland). ATMA-Br compounds were purchased from Aladdin (Shanghai, China) and H_2_O_2_ (30%) from Sinopharm (China). The chemical formulas of the ATMA compounds used in this study and their molecular weights are shown in Table 1. Each ATMA-Br compound was freshly prepared using a stock solution in distilled water.

*Microcystis* colonies were collected from a fish pond on 23 September 2020. Microscopic observation showed that *Microcystis viridis* and *Microcystis wesenbergii* were dominating. The geographical location of the fish pond and mesocosm set up are presented in Figure S1.

### 2.2. Laboratory Experiment

#### 2.2.1. Alkyl Chain Length and Inhibition of Photosynthesis in Microcystis Colonies

In this experiment, we assessed the effect of ATMAs with various alkyl chain lengths (from C12–C18) on *Microcystis* colonies collected from a fish pond. The colonies were diluted with 1/10 BG11 to a final chlorophyll *a* (Chl *a*) concentration of 300 μg/L medium in 25 cm^3^ culture flasks (Corning, New York, USA). Different doses of ATMA-Br compounds were added, and the suspensions were maintained at 25 °C under continuous light (100 μmol photons s^−1^ m^−2^ of cold fluorescent light) for 4 days. Photosynthetic parameters were measured by Handy plant efficiency analyzer (PEA, Hansatech, England), as described below (Section 2.4.2) at 3, 24 and 96 h post-exposure to ADTMA compounds.

The cyanocidal effect of ODTMA-Br (C18) was compared to that of H_2_O_2_, a commonly used cyanocide, applying the same experimental setup. The selected ODTMA-Br (C18) concentrations were 1, 2, 6.4 and 10 μM, whereas the H_2_O_2_ concentrations used were 36.8, 73.5, 147.1 and 294.1 μM. The photosynthetic activity (Fv/Fm) was measured 6, 24 and 96 h after each treatment.

#### 2.2.2. Exploring the Algaecidal Selectivity of ODTMA-Br (C18)

A total of 24 strains: 12 Cyanobacteria, 7 Chlorophyta, 2 Bacillariophyta, 2 Euglenozoa and 1 Cryptophyta were selected and cultured in six-well plates. Information on these strains is shown in Table 2. The concentrations of the ODTMA-Br (C18) applied were 0 (control), 2 and 4 μM. The photosynthetic parameters: Fo (initial fluorescence yield), and Fm (maximum fluorescence yield), were measured (after 15 min dark adaptation) at 6, 24 and 96 h post the addition of ODTMA-Br (C18) to the wells. The concentrations of Chl *a* were measured at 96 h. The Fv/Fm (maximum photochemical efficiency) values of the treatment group were compared with those of the control group, and the inhibition ratio of Fv/Fm (calculated by the following formula) was used to assess the ability of ODTMA-Br (C18) to inhibit algal photosynthesis.

(1) $$\mathrm{Inhibition}\;\mathrm{ratio}=\frac{\mathrm{Fv}/\mathrm{Fm}\;\left(\mathrm{Control}\;\mathrm{group}\right)-\mathrm{Fv}/\mathrm{Fm}\;\left(\mathrm{Treatment}\;\mathrm{group}\right)}{\mathrm{Fv}/\mathrm{Fm}\;\left(\mathrm{Control}\;\mathrm{group}\right)}$$

The higher the ratio, the larger the suppression effect.

### 2.3. Mesocosm Experiment

*Microcystis* colonies collected from a fish pond were first pooled in a 1500 L barrel for a day of acclimation. On day-0 of the mesocosm experiment, aliquots of the *Microcystis* colonies were placed into 200 L barrels with a final Chl *a* concentration of 300 μg/L. In order to simulate the original growth conditions of the collected colonies, the barrels were placed approximately 10 m from the fish pond (Figure S1). ODTMA-Br (C18) (6.4 μM) or H_2_O_2_ (117.6 μM) was added to the relevant barrels. Three untreated barrels were included as controls. The photosynthetic activity was measured on days 1, 3, 5, 7, 9, 13, 15 and 28 and the phytoplankton density and composition were analyzed on days 0, 1, 3, 5, 7, 9, 13, 15 and 28 after the addition of ODTMA-Br (C18) or H_2_O_2_.

### 2.4. Sample Analyses

#### 2.4.1. Measurement of Pigments Contents

Withdrawn cell suspensions (5 mL) were filtered through a Whatman GF/F membrane. Pigments were extracted in a 10 mL tube with 80% chilled acetone and kept in darkness for 12 h. Then, the tubes were centrifuged at 4 °C for 15 min at 6000× *g*. The optical densities of the extracts at 646, 663 and 750 nm were determined using a UV-VIS spectrophotometer (UV-1700, Shimadzu). The concentration of Chl *a* was calculated according to the method from Lichtenthaler and Wellburn [24].

#### 2.4.2. Measurement of Chl a Fluorescence Transient

To test the adverse effect on photosynthetic processes, the polyphasic Chl *a* fluorescence transient (the fluorescence induction-decay curve, also known as the Kautsky effect) was determined with a Handy PEA with an actinic light of 3000 μmol photos m^−2^ s^−2^ (excitation light, λ = 650 nm). The measuring light intensity is 10% of the actinic light (300 μmol photos m^−2^ s^−2^). All samples were dark acclimated for 15 min before measurement. The fluorescence signals were recorded within a time period from 10 μs to 2 s, and fluorescence kinetics showed a polyphasic rise over time, known as the O-J-I-P curve [25]. The initial fluorescence level O corresponds to the minimal Chl *a* fluorescence value (Fo). The J-I transient is caused by the gradual reduction of the primary electron acceptors, QA and QB. The P value is the maximal fluorescence yield (F_m_). The calculated parameters, based on the O-J-I-P fluorescence curve, are presented in Table S1 and as a radar plot in Figure S2.

#### 2.4.3. Determination of Phytoplankton Community

Phytoplankton samples (100 mL) from the mesocosm experiments were collected after mixing and immediately fixed with a 1% Lugol solution. The samples were quantified using a counting chamber (0.1 mL) under an optical microscope (Olympus CX41) at 400× magnification. Phytoplankton species were identified according to Hu [26]. The wet weight biomass of each species was calculated according to their morphometric characteristics [27].

### 2.5. Data Analyses

Basic statistical analyses were carried out using the SPSS 25.0 software. One-way analysis of variance (ANOVA) with a multiple post hoc (LSD) test (significance level is 0.05) was used to check the significant difference between the various treatments. All the data are presented as the mean of three replicates. Results visualization was performed using GraphPad Prism 8.0. The Bray–Curtis distance and PerMANOVA analyses were performed through package *vegan* using R (version 3.6.1).

## 3. Results and Discussion

### 3.1. The Effect of the Alkyl Chain Length on Photosynthetic Activity

**Figure 1:**
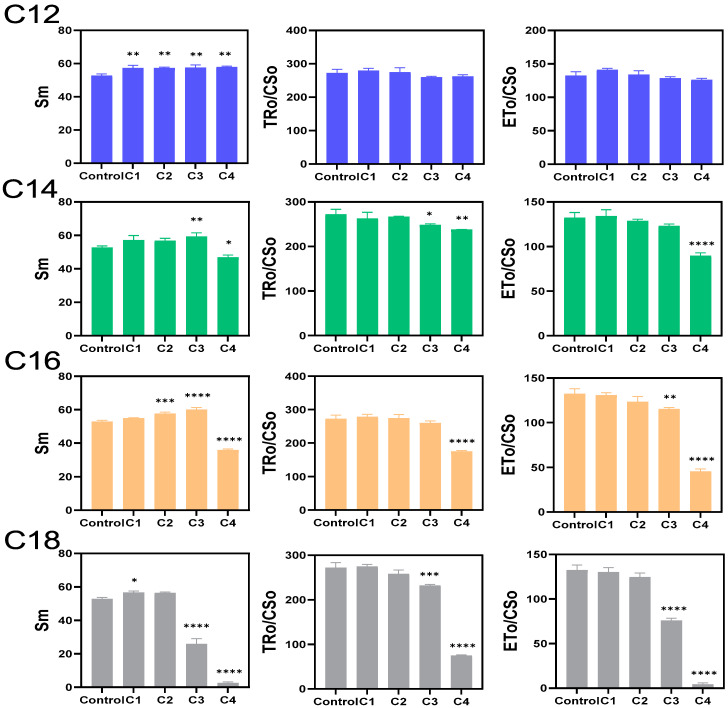
Effect of different ATMA-Br (C12–C18) and their concentrations (C1: 0.59 μM; C2: 1.78 μM; C3: 5.33 μM; C4: 16 μM) on three OJIP fluorescence parameters following 3 h exposure. Sm (the sum of area between multiple turn-overs of the OJIP curve) is calculated by the area between the OJIP curve and F = Fm line, indicating the total energy for receptor reduction. TR_O_/CS_O_ indicate the trapped energy flux per cross-section at t = 0) and ET_O_/CS_O_ indicates electron transport flux per cross-section at t = 0. A full presentation of OJIP fluorescence parameters was shown in Figure S2. Error bars indicate the standard deviation (n = 3). The asterisk indicates statistically significant differences in comparison with the control: * *p* < 0.05; ** *p* < 0.01; *** *p* < 0.005; **** *p* < 0.001.

It was reported that ATMA cations with longer alkyl chain exhibit stronger cyanocidal effect on unicellular *Microcystis* cultures [21]. To examine whether this is also the case with *Microcystis* colonies, the effectiveness of ATMA with alkyl chain length (C12 to C18) was examined using colonies collected in fish ponds. The photosynthetic parameters are shown in Figure 1 and Figure S2. After 3 h, ODTMA inhibited PSII activity stronger than the other ATMA compounds used. The PSII parameters Sm, TRo/CSo and ETo/CSo showed concentration-dependent decreases. The changes of these parameters indicated that the photochemical activity of *Microcystis* was suppressed, and the effective utilization rate of light energy decreased, resulting in the inhibition of photosynthesis. The alkyl chain length of ATMA refers to a biocidal effect towards different microorganisms. Gilbert and Moore [28] reported that molecular weight and N-alkyl chain length affect the efficacy of many QAC-based antimicrobial systems. The optimum alkyl chain length for killing yeast and filamentous fungi is C12, whereas it was C14 and C16 for gram-positive and gram-negative bacteria, respectively [28]. For cyanobacteria, Wu et al. [21] reported that the effect of ODTMA was strongest among ATMAs with an alkyl chain length from C10–C18. Similarly, to inactivate *Microcystis* colonies, ODTMA is more effective in our study.

#### 3.1.1. The Algaecidal Effect of ODTMA-Br (C18) on Cyanobacteria and Eukaryotic Algae

**Figure 2:**
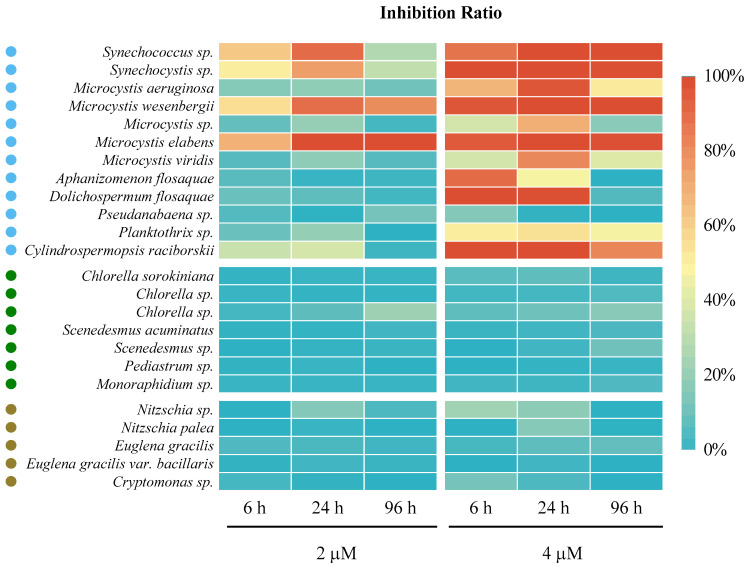
A heatmap presenting algaecidal spectrum and selectivity of ODTMA-Br (C18) at 2 and 4 μM. The inhibition ratio was calculated by the decrease in Fv/Fm value relative to control. A total of 24 cyanobacteria and eukaryotic algae, covering 12 Cyanobacteria (marked in blue cycles), 7 Chlorophyta (marked in green cycles) and 5 other eukaryotic algae (marked in brown cycles, including 2 Bacillariophyta, 2 Euglenozoa and 1 Cryptophyta) were used.

To investigate the algaecidal impact and selectivity of ODTMA-Br (C18) towards various cyanobacteria and eukaryotic algae species, we applied two ODTMA-Br (C18) concentrations to 24 species of algae that were commonly observed species in freshwater ecosystems, including fish ponds. Two μM of ODTMA-Br (C18) suppressed the activity of five different cyanobacteria within 6 and 24 h but doubling the concentration to 4 μM inhibited the activity of 12 different cyanobacteria (Figure 2). In contrast, the ODTMA-Br (C18) concentration used hardly affected the photosynthetic activities (Fv/Fm) of Chlorophyta, Bacillariophyta, Euglenozoa and Cryptophyta (Figure 2). One-way analysis of variance (ANOVA) with a multiple post hoc (LSD) test (significance level is 0.05) was used to check the significant difference between the various treatments. The results show that 6 and 24 h after a 4 μM ODTMA-Br (C18) treatment, there were significant differences between Cyanobacteria and Chlorophyta (*p* < 0.01), Cyanobacteria with Bacillariophyta, Euglenozoa and Cryptophyta (*p* < 0.01). These results suggest that when applied at a low concentration, ODTMA -Br (C18) can selectively inhibit cyanobacteria with minimal effect on algae. The reasons for the distinct sensitivity of cyanobacteria to ODTMA-Br (C18) treatment is not known but is likely related to differences in the composition of the cell wall and cell envelope, affecting the interaction with the ODTMA-Br (C18) cation and, consequently, the function and viability of the cells [29]. It is further postulated that the extent of damage to macromolecule within photosynthetic apparatus differs between cyanobacteria and algae, and hence the fluorescence parameters were much stronger affected in the former, confirming an earlier study [21,22]. Compared to eukaryotic algae, the cyanobacterial photosynthetic apparatus does not segregate into organelles and has a direct connection with the plasma membrane. Therefore, these differences in cell structure might result in a higher susceptibility of cyanobacteria to ODTMA-Br (C18).

#### 3.1.2. Laboratory-Scale Comparison between ODTMA-Br (C18) and H_2_O_2_ Efficacies as Cyanocides

**Figure 3:**
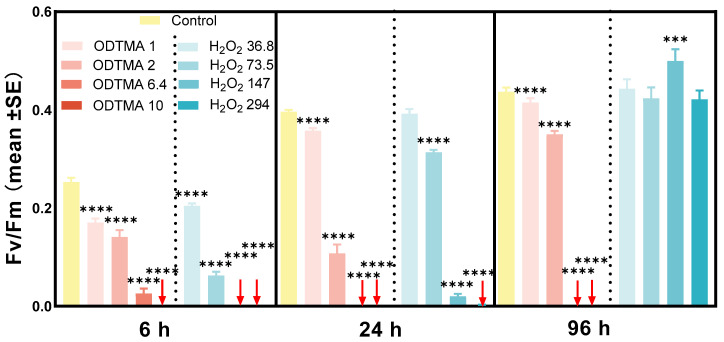
Photosynthetic activity changes (Fv/Fm) of site-collected *Microcystis* spp. colonies under ODTMA-Br (C18) and H_2_O_2_ treatments. Four concentrations of treatment were set for both ODTMA-Br (C18) (1, 2, 6.4 and 10 μM) and H_2_O_2_ (36.8, 73.5, 147 and 294 μM). The initial chl *a* concentration of each group was ca. 300 μg/L. Error bars indicate the standard deviation (n = 3). The asterisk indicates statistically significant differences in comparison with the control: ***, *p* < 0.005; ****, *p* < 0.001. Red arrows indicate the values of Fv/Fm were below the detection limit of the method.

The Fv/Fm value of ODTMA-Br (C18) or H_2_O_2_ treated cultures significantly decreased during the experiment (Figure 3). After 24 and 96 h, the Fv/Fm value was undetectable when a ODTMA-Br (C18) concentration higher than 6.4 μM was applied. Therefore, 6.4 μM of ODTMA-Br (C18) was used in the following mesocosm experiment. Contrary to the ODTMA-Br (C18) treatment, Fv/Fm increased significantly in 147 and 294 μM H_2_O_2_-treated cells after 96 h, suggesting that the photosynthetic activity of *Microcystis* might have recovered.

### 3.2. Mesocosm Experiment

To evaluate the cyanocidal performance of ODTMA-Br (C18) under field conditions, a mesocosm experiment was carried out where the effects of ODTMA-Br (C18) and H_2_O_2_ were compared.

#### 3.2.1. Morphological and Physiological Changes in Microcystis Colonies

*Microcystis* colonies were recorded prior to the addition of ODTMA-Br (C18) or H_2_O_2_ (day 0), depicting neatly arranged cells embedded in a gelatinous matrix (Figure S3). Three days post-exposure to either ODTMA-Br (C18) or H_2_O_2_, the *Microcystis* colonies disintegrated, and their pigments degraded (Figure S3). The morphological changes 9 days post-treatment indicated that the majority of *Microcystis* colonies exposed to either ODTMA-Br (C18) or H_2_O_2_ had been effectively disintegrated and disappeared. The green algae *Scenedesmus* sp. thrived in both ODTMA-Br (C18) and H_2_O_2_-treated groups throughout the mesocosm experiment (Figure S3 day 28).

**Figure 4:**
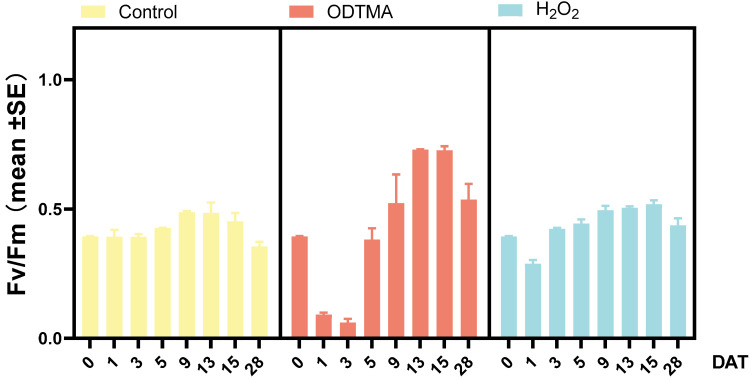
Temporal variations in photosynthetic activity (Fv/Fm) measured in *Microcystis* cultures maintained in mesocosms and exposed to a single dose of ODTMA-Br (C18) (6.4 μM) or H_2_O_2_ (117 μM).

**Figure 5:**
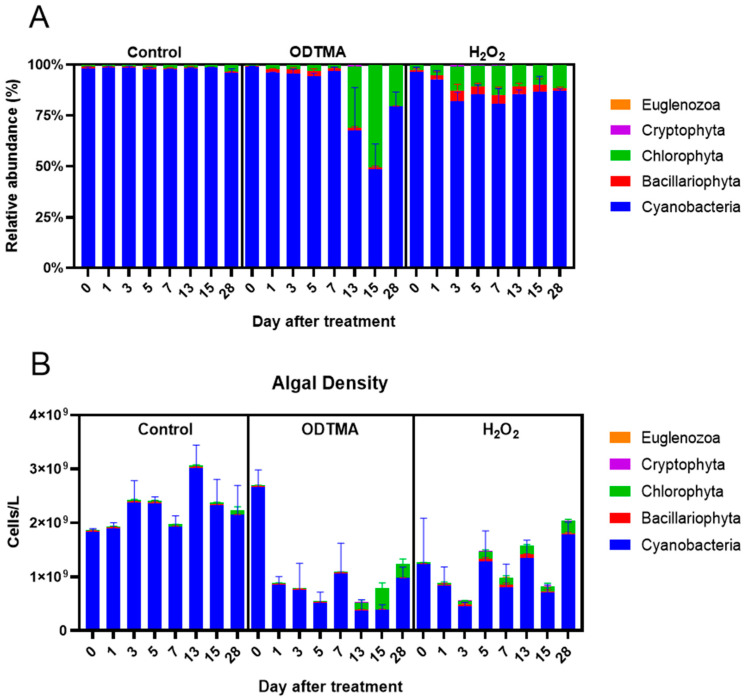
Population dynamics of phytoplankton community during the mesocosm experiment. (**A**) The relative abundance of major Phyla; (**B**) cell density of cyanobacteria, Chlorophyta, diatom, Euglenozoa and Cryptophyta. The concentrations of ODTMA -Br (C18) and H_2_O_2_ treatments were 6.4 and 117 μM, respectively.

Fv/Fm declined rapidly during day 1 and increased from day 3 in both ODTMA-Br (C18) and H_2_O_2_ treatments (Figure 4). From day 13, the Fv/Fm values increased markedly in ODTMA-Br (C18) treated mesocosms to ca. 0.7, which is commonly obtained in green algae but not *Microcystis* cells. The rise in Fv/Fm agrees with the microscopic observation (Figure S3) that indicated a significant increase of the cell count of *Scenedesmus* sp. at the end of the mesocosm experiment (Figure 5).

Similar results have been reported in several other studies where H_2_O_2_ was used as cyanocide. Weenink and colleagues [13] proposed that competition by green algae restrained the growth of cyanobacteria in H_2_O_2_-treated waters. In a field experiment, Yang and co-authors [30] found that H_2_O_2_ treatment could promote the growth of chlorophytes, and the dominant genera were *Coelastrum, Pediastrum, Scenedesmus* and *Staurastrum*. Wang et al. [31] reported that *Chlamydomonas* sp. thrived after suppression of the *Microcystis* bloom.

#### 3.2.2. Impacts on the Long-Term Biodiversity

**Figure 6:**
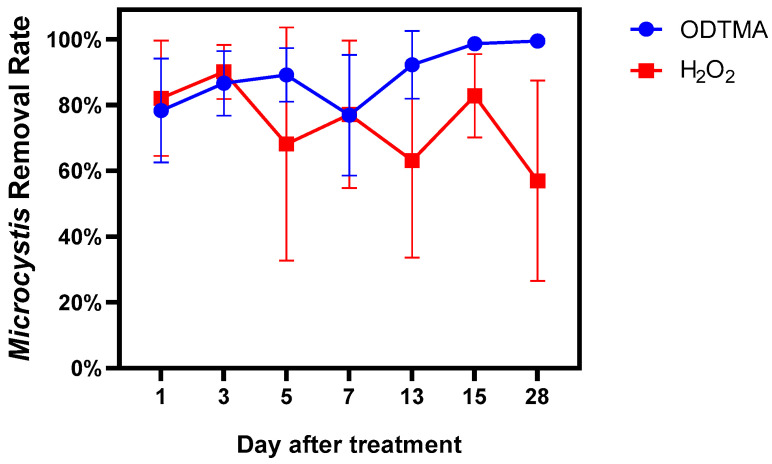
Removal rate of *Microcystis* cells during the 28-day mesocosm experiment. The initial cell density of *Microcystis* sp. was 1.83 × 10^6^ cells/mL.

Figure 5A shows the proportion of the major phyla found in the mesocosms. Cyanobacteria accounted for more than 90% of the phytoplankton population in the untreated controls during the entire experiment. The cell densities of Cyanobacteria were 1.83 × 10^9^ and 2.14 × 10^9^ cells/L on days 0 and 28, respectively, in the untreated controls (Figure 5B). The addition of ODTMA-Br (C18) or H_2_O_2_ lowered the cyanobacteria cell count but raised the abundance of eukaryotic algae. These responses of the mesocosm population to H_2_O_2_ treatment are compatible with several studies showing that H_2_O_2_ can reduce the abundance of cyanobacteria relative to eukaryotic algae [32,33]. Total algal density in the ODTMA-Br (C18) group dropped to the lowest level on day 5, and the proportion of Chlorophyta was significantly higher on day 13. On day 15, Chlorophyta almost overtook that of Cyanobacteria as the dominant species (Figure 5A). In the H_2_O_2_ treatment, the cell density of Cyanobacteria dropped to the lowest level on day 3, the biomass of Bacillariophyta increased on day 1 and that of Chlorophyta increased on day 3. However, there was a significant rise in cyanobacterial biomass from day 5 in the H_2_O_2_ group. In terms of the duration of the cyanocidal effect, at the end of the experiment (day 28), despite the presence of a significant count of green algae in the H_2_O_2_ group, the cyanobacteria recovered to the original level on day 0. In contrast, cyanobacterial biomass in the ODTMA-Br (C18) group was reduced by nearly 50% compared to day 0. The removal rate of *Microcystis* cells (Figure 6) shows that the difference between ODTMA-Br (C18) and H_2_O_2_ treatments became distinct from day 7. As an example, on day 28, the *Microcystis* removal rate in ODTMA-Br (C18) treated mesocosms was 99. 5%, whereas it was only 57.0% after H_2_O_2_.

Currently, one of the major drawbacks of using algaecide for cyanobacterial bloom control is their limit in longevity, an important criterion in the selection of the proper cyanocide to be applied [5]. Most likely, the difference between the two cyanocides used here relates to the fact that H_2_O_2_ is rapidly degraded by biological and physicochemical processes, whereas the ODTMA-Br (C18), which stays in the aqueous system as micelles or by binding to solid particles, is far more stable [14].

At present, it appears that the variability in H_2_O_2_ efficacy depends on the type and density of cyanobacteria present, the water chemistry and the isolation of the water body [15]. In many cases, repeated application of H_2_O_2_ is employed to control cyanobacterial blooms during an entire season. A recent study reported that two consecutive applications of a low H_2_O_2_ dose were much more efficient against *M. aeruginosa* strain MGK than a single treatment with a higher H_2_O_2_ concentration [34].

**Figure 7:**
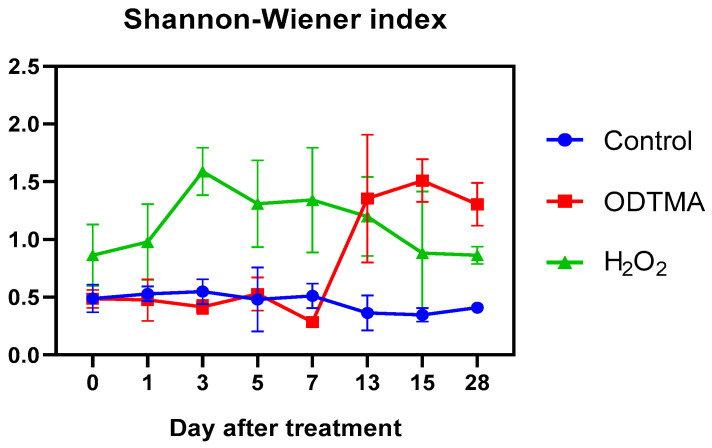
Species diversity indices (Shannon–Wiener) for ODTMA-Br (C18) and H_2_O_2_ treatment in the mesocosm experiment.

The biodiversity reflects the compositional characteristics and function of a community. Increased biodiversity indicates more complicated relationships among species and food web structures, which can result in the community having a greater resilience and stability in response to external environmental fluctuations [35]. The outbreak of cyanobacterial blooms reduces biodiversity and causes damage to ecological functions [36]. The species diversity index of the phytoplankton community in the mesocosms gradually increased following the different algaecide treatments (Figure 7). A marked rise in the Shannon–Wiener index was already observed 7 days after the H_2_O_2_ treatment, and the maximum value reached was more than three times higher than the control, but then gradually decreased to 1.0 by the end of the experiment. At that time, after ODTMA-Br (C18) application, the biodiversity was 3.2 times higher than in the control and 1.5 times that of the H_2_O_2_ treatment. Given the longer exposure to ODTMA-Br (C18) compared with H_2_O_2_ (mentioned above), our results show that ODTMA-Br (C18) has an advantage over H_2_O_2_ in the long-term impact on the biodiversity. A similar conclusion is drawn from a Bray–Curtis distance of dissimilarity (Figure S4) and PerMANOVA analyses (Table S2). The dissimilarity coefficients of the Bray–Curtis distance between ODTMA-Br (C18) and control groups was above 0.4 during the 28-day experiment, suggesting that the composition of the phytoplankton community is distinct between the ODTMA-Br (C18) and control groups. Similarly, the composition of the phytoplankton community of the ODTMA -Br (C18) group differed from the H_2_O_2_ group (dissimilarity coefficient > 0.4). However, the dissimilarity coefficient between the H_2_O_2_ and control groups were above 0.4 by day 15 and below 0.4 on day 28, indicating the recovery of *Microcystis.* PerMANOVA, based on the phytoplankton cell density and biomass throughout the mesocosm experiment, showed that there were significant differences in the total phytoplankton composition and Diatom composition of both ODTMA-Br (C18) and H_2_O_2_ treatments compared to those in the control (*p* < 0.05, Table S2). The difference in Chlorophyte composition was significant between the control and H_2_O_2_ treated groups, while it was not significant between the control and ODTMA-Br (C18) groups.

The dynamics of the phytoplankton population in the mesocosm experiment was reflected in the rise in the eukaryotic algae proportion, especially green algae and diatoms, in response to the application of the cyanocides, likely because they are less susceptible to ODTMA-Br (C18) or H_2_O_2_ than cyanobacteria [37]. In the whole lake experiment, the selective damage of H_2_O_2_ to cyanobacteria was further aggravated [11,38]. The specific advantage of H_2_O_2_ is more significant when two applications of a low concentration of H_2_O_2_ are applied [5,34]. Meanwhile, ODTMA-Br (C18) exerts a selective effect on cyanobacteria, green algae and diatoms [21]. The data obtained in this study suggested that ODTMA-Br (C18) might be a cyanocide candidate with a relatively longer duration in suppressing cyanobacterial blooms. Although several studies have reported the (eco)toxicity of QACs [39,40], these compounds are adsorbed into particulate matter rapidly after application [41,42], and the possibility to become bioavailable through leaching from the particulate fraction is very low at environmentally relevant concentrations [43]. Moreover, QACs are susceptible to microbial degradation [44]. In practice, further clarification/assessment of the ecological “fingerprint”, impact, fate and risk of ODTMA-Br (C18) treatments are requested before application in the field.

## 4. Conclusions

The current study showed that ODTMA-Br (C18) is an efficient and selective cyanocide with good application prospects to control and manage harmful *Microcystis* blooms in fish ponds. Compared with a single low dose of H_2_O_2_ application, a single dose of ODTMA-Br (C18) had a more persistent inactivation of *Microcystis* colonies under the tested conditions. Moreover, the price nowadays of ODTMA-Br (Br) is fourfold higher than H_2_O_2_. Though QACs have been widely used as cationic disinfectants and surfactants, their direct application in field-controlling cyanobacterial blooms has yet to be evaluated and documented.

## Figures and Tables

**Table 1:** Chemical properties of the four ATMA-Br used in this study.

Acronyms of ATMA Bromides	Chemical Formula	MW (g/mol)
ODTMA—Br (C18)	CH3(CH2)17N(Br)(CH3)	392.51
HDTMA—Br (C16)	CH3(CH2)15N(Br)(CH3)	364.45
TDTMA—Br (C14)	CH3(CH2)13N(Br)(CH3)	336.40
DDTMA—Br (C12)	CH3(CH2)11N(Br)(CH3)	280.29

**Table 2:** Cyanobacteria and eukaryotic algae used in this study.

	Number	Genus	Species	Culture Medium
1	FACHB805	*Synechococcus*	*Synechococcus* sp.	BG11
2	PCC6803	*Synechocystis*	*Synechocystis* sp.	BG11
3	FACHB905	*Microcystis*	*Microcystis aeruginosa*	BG11
4	FACHB908	*Microcystis*	*Microcystis wesenbergii*	BG11
5	FACHB915	*Microcystis*	*Microcystis* sp.	BG11
6	FACHB917	*Microcystis*	*Microcystis elabens*	BG11
7	FACHB979	*Microcystis*	*Microcystis viridis*	BG11
8	FACHB1171	*Aphanizomenon*	*Aphanizomenon flos-aquae*	BG11
9	FACHB1255	*Dolichospermum*	*Dolichospermum flos-aquae*	BG11
10	FACHB1277	*Pseudanabaena*	*Pseudanabaena* sp.	BG11
11	FACHB1365	*Planktothrix*	*Planktothrix* sp.	BG11
12	FACHB1503	*Cylindrospermopsis*	*Cylindrospermopsis raciborskii*	BG11
13	FACHB26	*Chlorella*	*Chlorella sorokiniana*	BG11
14	FACHB1552	*Chlorella*	*Chlorella* sp.	BG11
15	FACHB1580	*Chlorella*	*Chlorella* sp.	BG11
16	FACHB1235	*Scenedesmus*	*Scenedesmus acuminatus*	BG11
17	FACHB2944	*Scenedesmus*	*Scenedesmus* sp.	BG11
18	FACHB2945	*Pediastrum*	*Pediastrum* sp.	BG11
19	FACHB2952	*Monoraphidium*	*Monoraphidium* sp.	BG11
20	FACHB512	*Nitzschia*	*Nitzschia* sp.	CSI
21	FACHB2935	*Nitzschia*	*Nitzschia palea*	CSI
22	FACHB848	*Euglena*	*Euglena gracilis*	HUT
23	FACHB850	*Euglena*	*Euglena gracilis var. bacillaris*	HUT
24	FACHB1943	*Cryptomonas*	*Cryptomonas* sp.	AF-6

## Data Availability

Not applicable.

## Supplementary Materials

The following are available online at https://www.mdpi.com/article/10.3390/microorganisms9112258/s1, Table S1. JIP test parameters with explanations and equation calculated using data extracted from the O-J-I-P fast Figure 1995. Table S2. PerMANOVA assessment of the differences in total phytoplankton, Chlorophyte, and Diatom communities between every two treatments based on biomass during the 28 days experiment. Figure S1. Photos of the fish pond where Microcystis spp. colonies were collected and site of the mesocosm experiment. The geographic location is 30.53N 114.40E. Figure S2. The photosynthetic parameters measured in Microcystis colonies exposed to different ATMA-Br for 3 h. Figure S3. ODTMA and H2O2 impose morphological and physiological changes in Microcystis colonies. Figure S4. Bray-Curtis distance and Metric multidimensional scaling analyses between the control, ODTMA, and H2O2 groups. Dissimilarity coefficients (a value greater than 0.4 indicates that there was a difference in species composition between the two treatments.
