# Supplementary material for: Can Alkyl Quaternary Ammonium Cations Substitute H2O2 in Controlling Cyanobacterial Blooms—Laboratory and Mesocosm Studies
Source: Microorganisms. 2021 Oct 29;9(11):2258. doi: 10.3390/microorganisms9112258 (PMC8619391; doi:10.3390/microorganisms9112258)
Supplement: Supplementary file 1 [file microorganisms-09-02258-s001.zip › microorganisms-1342259-supplementary.pdf]

microorganisms

MDPI

Supplementary materials

Table S1. JIP test parameters with explanations and equation calculated using data extracted from the O-J-I-P fast Figure 1995.

	PJIP test parameters	Explanation
1	F_o	Minimum fluorescence intensity
2	Fv/Fm=(Fm-Fo)/Fm	maximum quantum yield of primary PSII photochemistry after dark adaptation
3	Sm = Area/(Fm – Fo)	y-axis and the area between the fluorescence intensities (F = Fm)
4	TRo/CSo = φPo·(ABS/CSo)	Trapped energy flux per CS (at t = 0)
5	ETo/CSo = φ_{Eo}·(ABS/CSo)	Electron transport flux per CS (at t = 0)
6	V_J= (F_{2ms} – Fo)/(Fm – Fo)	Relative variable fluorescence intensity at the J-step, reflecting the open state of reaction centers
7	M_0= 4 ($F_{300\mu s}$–Fo)/(F_m–Fo)	Approximated initial slope of the fluorescence transient, reflecting the maximum speed of Q_A reduction
8	φ_{Eo} =ETo/ABS= [1-(Fo/Fm)] •ψ_0	Quantum yield for electron transport (at t = 0)
9	ψ_0=ET_O/TR_O = (1 –V_J)	Probability that a trapped excition transfers an electron into the electron transport chain beyond Q_A (at t = 0)
10	DIo/CSo = ABS/CSo – TRo/CSo	Dissipated energy flux per CS (at t = 0)
11	RC/CSo=φ_{Po}•(V_J/Mo)•(ABS/CSo)	Number of RCs per CS, reflecting density of RCs
12	ABS/RC = Mo·(1/V_J)·(1/φ_{Po})	Absorption flux per reaction center (RC)
13	ETo/RC = Mo·(1/V_J)·Ψo	Electron transport flux per RC (at t = 0)
14	DIo/RC = ABS/RC – TRo/RC	Dissipated energy flux per RC (at t = 0)
15	TRo/RC = Mo·(1/V_J)	Trapped energy flux per RC (at t = 0)
16	RC/ABS = (1/ Mo)·φ_{Po} ·V_J	Density of RCs based on absorbed energy
17	PIABS = (RC/ABS)·[φ_{Po}/(1 – φ_{Po})]·[Ψo/(1 – Ψo)]	Performance index based on absorption of light energy
18	Phi(Po)	Maximum photochemical efficiency
19	ABS/CSo = Mo·(1/V_J)·(1/φ_{Po})	Absorption flux per reaction center (RC)
20	DIo/CSo = ABS/CSo – TRo/CSo	Dissipated energy flux per CS (at t = 0)
21	REo/CSo	Energy transferred to the end of the electron transfer chain per CS (t=0)

Table S2. PerMANOVA assessment of the differences in total phytoplankton, Chlorophyte, and Diatom communities between every two treatments based on biomass during the 28 days experiment.

	total phytoplankton		chlorophyte		diatom	
pairs	R^2	P value	R^2	P value	R^2	P value
ODTMA vs H_2O_2	0.0435	0.183	0.0745	0.003*	0.0700	0.009*
ODTMA vs Ctrl	0.0931	0.009*	0.0366	0.207	0.0729	0.021*
H_2O_2 vs Ctrl	0.0863	0.042*	0.0509	0.027*	0.1256	0.003*

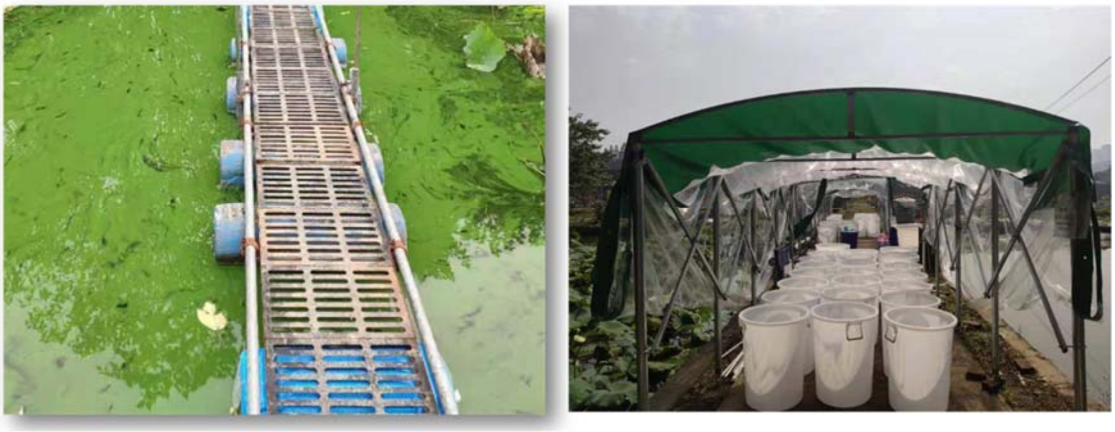

Figure S1. Photos of the fish pond where Microcystis spp. colonies were collected and site of the mesocosm experiment. The geographic location is 30.53N 114.40E.

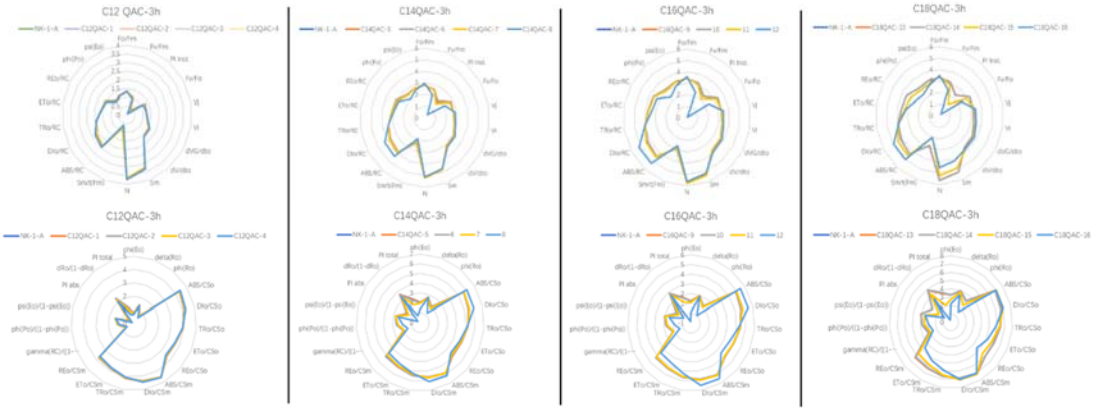

Figure S2. The photosynthetic parameters measured in Microcystis colonies exposed to different ATMA-Br for 3 hours.

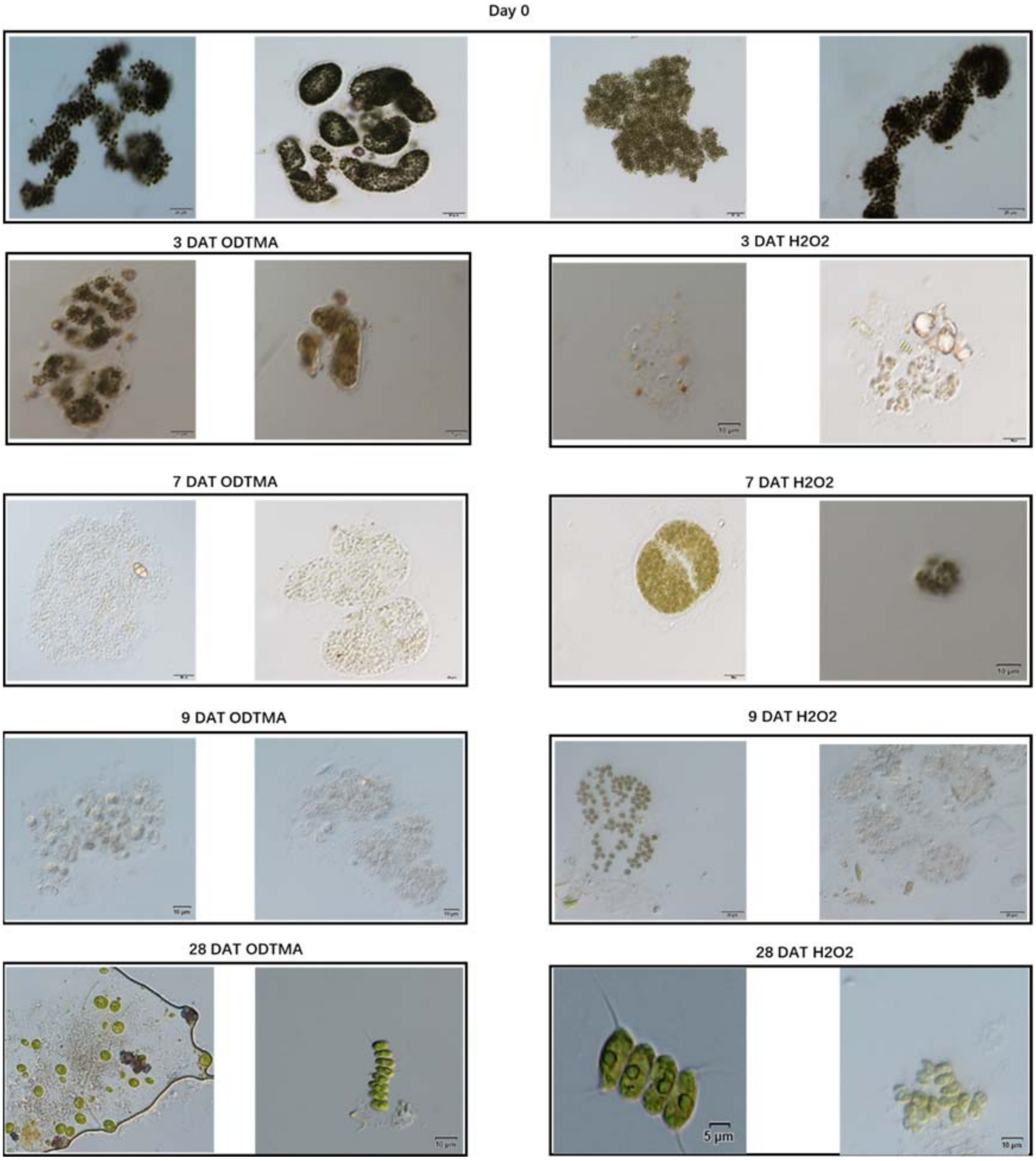

Figure S3. ODTMA and H2O2 impose morphological and physiological changes in Microcystis colonies.

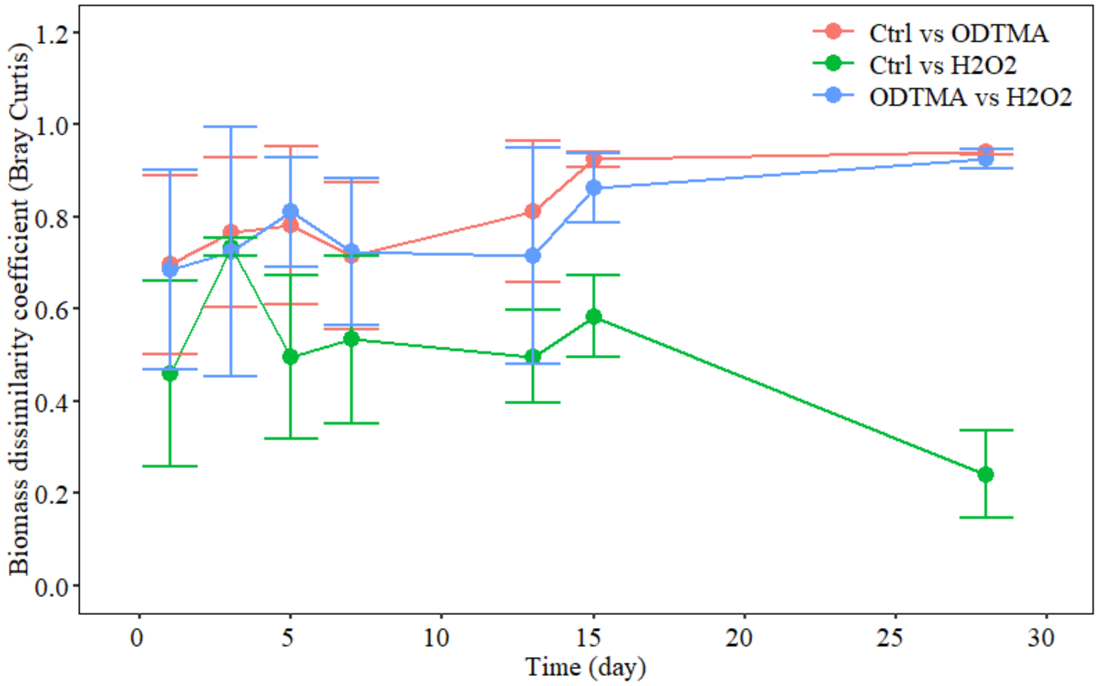

Figure S4. Bray-Curtis distance and Metric multidimensional scaling analyses between the control, ODTMA, and H2O2 groups. Dissimilarity coefficients (a value greater than 0.4 indicates that there was a difference in species composition between the two treatments.
